# Supplementary material for: Multi‐dataset identification of innovative feature genes and molecular mechanisms in keratoconus
Source: J Cell Mol Med. 2024 Sep 19;28(18):e70079. doi: 10.1111/jcmm.70079 (PMC11412914; doi:10.1111/jcmm.70079)
Supplement: Supplementary file 2 — Material S2. [file JCMM-28-e70079-s002.zip › 7.GSEA analysis/1.ARL11/KEGG_PROXIMAL_TUBULE_BICARBONATE_RECLAMATION.html]

Details for gene set KEGG\_PROXIMAL\_TUBULE\_BICARBONATE\_RECLAMATION[GSEA]

|  || Dataset | ARL11.group.cls#h\_versus\_l |
| Phenotype | group.cls#h\_versus\_l |
| Upregulated in class | l |
| GeneSet | KEGG\_PROXIMAL\_TUBULE\_BICARBONATE\_RECLAMATION |
| Enrichment Score (ES) | -0.30175865 |
| Normalized Enrichment Score (NES) | -0.9177083 |
| Nominal p-value | 0.61728394 |
| FDR q-value | 1.0 |
| FWER p-Value | 1.0 |
Table: GSEA Results Summary

  

Fig 1: Enrichment plot: KEGG\_PROXIMAL\_TUBULE\_BICARBONATE\_RECLAMATION      
 Profile of the Running ES Score & Positions of GeneSet Members on the Rank Ordered List

  

| SYMBOL | TITLE | RANK IN GENE LIST | RANK METRIC SCORE | RUNNING ES | CORE ENRICHMENT || 1 | SLC9A3 | na | 2239 | 0.280 | 0.0432 | No |
| 2 | ATP1B4 | na | 2566 | 0.261 | 0.1343 | No |
| 3 | PCK1 | na | 5840 | 0.141 | 0.0943 | No |
| 4 | ATP1B1 | na | 6792 | 0.117 | 0.1118 | No |
| 5 | GLS2 | na | 11078 | 0.047 | 0.0063 | No |
| 6 | GLUD1 | na | 12533 | 0.031 | -0.0237 | No |
| 7 | ATP1A1 | na | 13911 | 0.020 | -0.0558 | No |
| 8 | SLC25A10 | na | 14007 | 0.019 | -0.0514 | No |
| 9 | MDH1 | na | 18516 | -0.014 | -0.1760 | No |
| 10 | ATP1B3 | na | 19840 | -0.023 | -0.2054 | No |
| 11 | ATP1A3 | na | 20328 | -0.026 | -0.2093 | No |
| 12 | GLS | na | 21462 | -0.035 | -0.2285 | No |
| 13 | FXYD2 | na | 22512 | -0.045 | -0.2414 | No |
| 14 | ATP1A4 | na | 22955 | -0.049 | -0.2351 | No |
| 15 | SLC38A3 | na | 23032 | -0.050 | -0.2178 | No |
| 16 | ATP1B2 | na | 23314 | -0.054 | -0.2053 | No |
| 17 | GLUD2 | na | 26662 | -0.102 | -0.2626 | Yes |
| 18 | AQP1 | na | 27073 | -0.110 | -0.2322 | Yes |
| 19 | SLC4A4 | na | 28594 | -0.146 | -0.2197 | Yes |
| 20 | CA4 | na | 28983 | -0.156 | -0.1709 | Yes |
| 21 | ATP1A2 | na | 29374 | -0.168 | -0.1175 | Yes |
| 22 | PCK2 | na | 30472 | -0.203 | -0.0709 | Yes |
| 23 | CA2 | na | 34417 | -0.503 | 0.0090 | Yes |
Table: GSEA details [plain text format]

  

Fig 2: KEGG\_PROXIMAL\_TUBULE\_BICARBONATE\_RECLAMATION      
 Blue-Pink O' Gram in the Space of the Analyzed GeneSet

  

Fig 3: KEGG\_PROXIMAL\_TUBULE\_BICARBONATE\_RECLAMATION: Random ES distribution      
 Gene set null distribution of ES for **KEGG\_PROXIMAL\_TUBULE\_BICARBONATE\_RECLAMATION**
